# Use of metagenomic next-generation sequencing for diagnosis of peritonitis in end-stage liver disease

**DOI:** 10.7150/ijms.89242

**Published:** 2023-10-09

**Authors:** Taiyu He, Ning Luo, Juan Kang, Ning Ling, Dazhi Zhang

**Affiliations:** Department of Infectious Diseases, Key Laboratory of Molecular Biology for Infectious Diseases (Ministry of Education), Institute for Viral Hepatitis, the Second Affiliated Hospital, Chongqing Medical University, Chongqing, China.

**Keywords:** Metagenomic next-generation sequencing, conventional methods, peritonitis, end-stage liver disease, diagnosis

## Abstract

**Background:** Conventional methods are low in positive rates and time-consuming for ascites pathogen detection in patients with end-stage liver disease (ESLD). With many advantages, metagenomic next-generation sequencing (mNGS) may be a good alternative method. However, the related studies are still lacking.

**Methods:** Ascites from 50 ESLD patients were sampled for pathogen detection using mNGS and conventional methods (culture and polymorphonuclear neutrophils detection) in this prospective observational study.

**Results:** Forty-two samples were detected positive using mNGS. 29 strains of bacteria, 11 strains of fungi, and 9 strains of viruses were detected. 46% of patients were detected to be co-infected with 2 or more pathogens by mNGS. Moreover, mNGS showed similar and high positive rates in ESLD patients with different clinical characteristics. Compared to conventional methods, mNGS had higher positivity rates (84% vs. 20%, *P*<0.001), sensitivity (45.2% vs. 23.8%, *P*=0.039), broader pathogen spectrum, shorter detection time (24 hours vs. 3-7 days), but lower specificity (25% vs 100%, *P* = 0.010). Furthermore, compared to conventional methods, mNGS showed similar consistence with final diagnosis (42% vs. 36%, *P*=0.539).

**Conclusions:** mNGS may be a good supplement for conventional methods and helpful to early etiological diagnosis of peritonitis, and thus improve ESLD patients' survival.

## Introduction

End-stage liver disease (ESLD) is the advanced stage of liver disease caused by various chronic liver damage. The mortality in patients with ESLD is high, and the number of ESLD patients has been increasing in recent years 1, 2. Peritonitis is a common complication in ESLD patients, which significantly increases their mortality 3-7. Therefore, accurate and early diagnosis and treatment of peritonitis is of great importance for improving the prognosis of ESLD patients.

However, patients with peritonitis often have insidious onset without specific clinical symptoms (such as fever, abdominal tenderness), and one-third of patients can be asymptomatic 7. Moreover, the ascitic fluid culture are low in positive rates and time-consuming (3 to 7 days), which hinders the early diagnosis (especially the early etiological diagnosis) of peritonitis 6-8. Bacteria, especially gram-negative bacteria, were commonly identified pathogens through ascites culture 9, but some pathogens, especially rare pathogens, are difficult to be detected through ascites culture, which affects the diagnosis and treatment on peritonitis caused by the latter pathogens. Currently in clinical practice, an ascitic neutrophil count ≥ 250/μL is often considered of peritonitis 6, 7, 10. And if so (ascitic neutrophil count ≥ 250/μL), even in the absence of an identified pathogen, empiric antibiotic therapy will be usually initiated. But this may increase the risk of drug resistance and the development of secondary infections 6, 7, 10, and thus worsen prognosis. Therefore, to deal with peritonitis more effectively, an accurate and rapid diagnostic method for identifying pathogens is urgently needed.

Metagenomic next-generation sequencing (mNGS) is an emerging etiological diagnostic technology that enables rapid detection of all potential pathogens 11. This technology is currently used for the etiological diagnosis of various infectious diseases such as central nervous system infections, blood infections and lung infections, and has shown good accuracy 11-13. However, the studies focusing on the use of mNGS for the etiological diagnosis of peritonitis in ESLD patients are still lacking.

Therefore, this study prospectively observed 50 ESLD patients and collected their ascites samples for pathogen detection using mNGS and clinical conventional methods (culture and polymorphonuclear neutrophils detection). To assess the clinical value of mNGS in the early etiological diagnosis of peritonitis in ESLD patients, the results of mNGS were compared with those of clinical conventional methods.

## Materials and Methods

### Patient inclusion

From May 10, 2022, to August 8, 2022, 50 adult patients with clinically-confirmed ESLD were enrolled in this prospective observational study at the Second Affiliated Hospital of Chongqing Medical University. The inclusion criteria for patients were as follows: 1) with ascites; 2) not yet diagnosed as peritonitis before inclusion. Demographic and clinical data were obtained using electronic medical records. Ascites samples were collected from all participants before antibiotic therapy by medical professionals following the principle of aseptic technique for pathogen detection, and the ascites culture was conducted by the department of the clinical laboratory of the Second Affiliated Hospital of Chongqing Medical University. All 50 patients were prospectively followed up till their final clinical outcomes.

This study was approved by the ethics committee of the Second Affiliated Hospital of Chongqing Medical University and registered at www.chictr.org.cn (ChiCTR2200059689) and conducted in accordance with both the Declarations of Helsinki and Istanbul. Written informed consent was obtained from all patients prior to enrollment.

### Ascites pathogen detection using metagenomic next-generation sequencing

Immediately after collection, ascites samples were transported on dry ice to Chongqing KingMed Diagnostics (China) for pathogen detection using mNGS. DNA was extracted from all samples with a Genskey Micro DNA Kit (1901, Genskey, China) following the manufacturer's manual, and the total mass of extracted DNA was measured by Qubit dsDNA HS Assay Kits (Q32854, Thermo Fisher Scientific, USA). DNA libraries were then built using Sequencing Reaction Universal Kit (Genskey, China) following the manufacturer's manual. For quality evaluation of DNA libraries, the concentration of the constructed DNA library was determined by qPCR using Applied BiosystemsTM 7500 (Thermo Fisher Scientific) and should be more than 1 Nmol/L. Then, DNA libraries with high quality were sequenced on a NextSeq CN 500 platform (Illumina, Inc., USA) using a High Output Reagent Kit (sequencing read length = SE75). Each sequencing ran along with a positive control and a negative control. Bcl2fastq software (version 2.20.0.422) was used to split BCL sequence data into fastq-format files for each sample, and the raw reads were quality filtered using fastp (version 0.23.2) and kz software (version 0.3.6), which removed adapter contamination and low-quality and low-complexity reads. The human DNA was filtered out after aligning to a human reference genome database (hg38). And the remaining reads were then aligned to the NCBI RefSeq (microbial) genome database. For reliability, if the number of reads detected for a species was less than 3, the read sequences of the corresponding species would be aligned to the NT database for verification. For all the pathogen originally detected, the obvious sequence alignment abnormalities (for the detected species, genome coverage < 1% and depth < 2) and background polluted bacteria (considered to be the exact background bacteria when the taxon-specific read number falls within the normal fluctuation range of historical statistical data compared with the negative controls) were first filtered out, and then pathogen data interpretation and pathogen positive determination were carried out.

### Statistical analysis

The Mann-Whitney U test was used for continuous-variable comparisons. The chi-square test and Fisher's exact test were used for the categorical-variable comparisons. Kendall's rank correlation and multivariate linear regression were performed for correlation analyses. A two-sided *P* value < 0.05 was considered of statistical significance. SPSS (version 24.0.0) was used for statistical analysis and GraphPad Prism (version 9.2.0) for plotting.

## Results

### Patient characteristics

Patients' characteristics are summarized in Table 1. The median age of ESLD patients was 56.0 years (range 18.0-76.0 years), and the median body mass index (BMI) was 22.2 kg/m^2 (range 14.8-34.9 kg/m^2). Among the 50 patients, 70% of them were male. Etiologies of ESLD in 58% of patients were hepatitis B virus infection.

### Pathogen detection using mNGS and ascites culture

**Figure 1:**
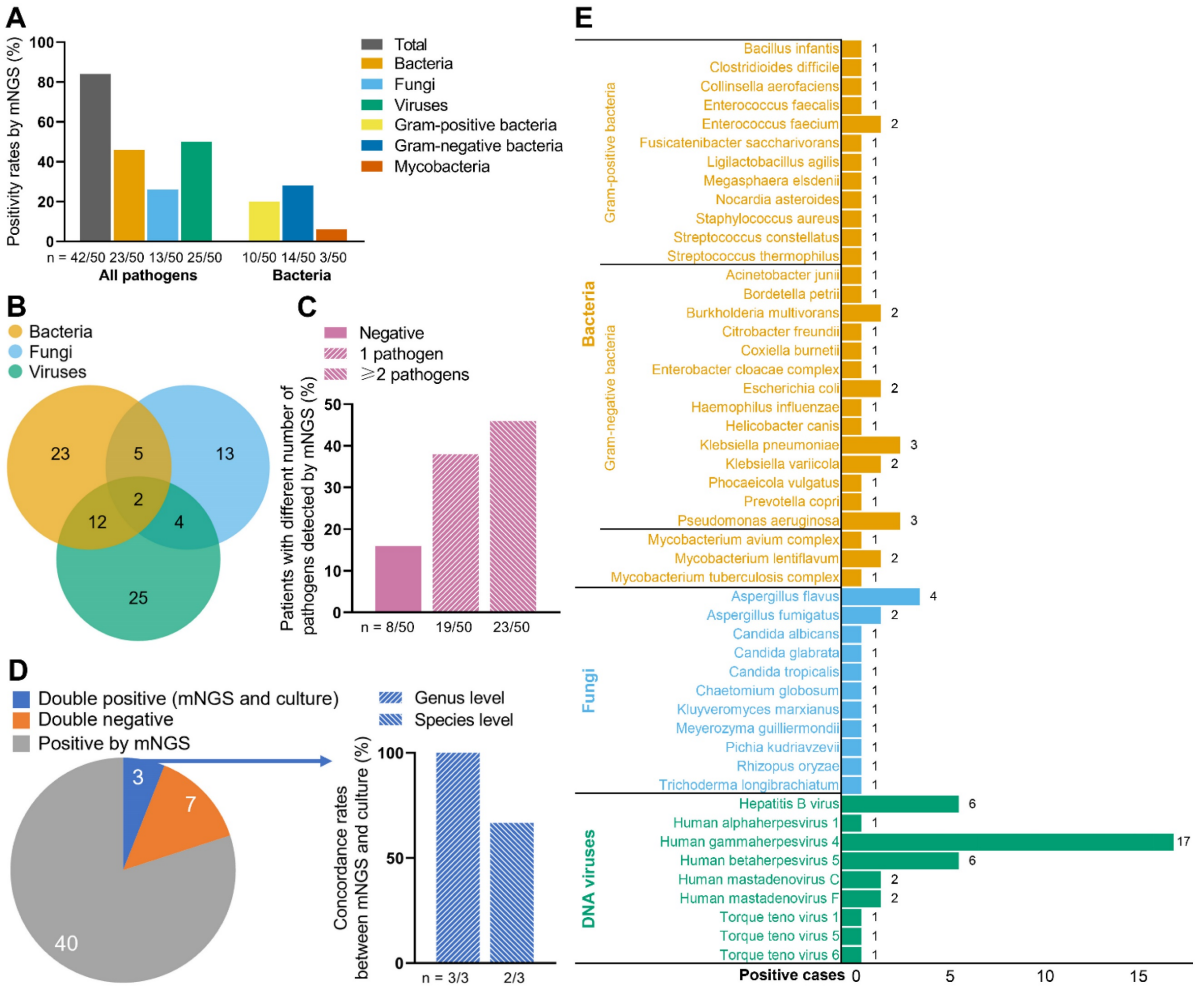
** Pathogen detection by mNGS and ascites culture. (A)** Positive rates of all pathogens and bacteria by mNGS in the 50 ESLD patients. **(B)** Numbers of patients detected positive for bacteria/fungi/viruses by mNGS. **(C)** Patients with different numbers of pathogens detected by mNGS. **(D)** Concordance between mNGS and ascites culture. **(E)** Positive cases of bacteria, fungi, and DNA viruses. mNGS, metagenomic next-generation sequencing. Notes: when the ascites culture and the supplementary results of mNGS were both positive, the sample were also defined as double positive.

Figure 1 demonstrates the results of the 50 ascites samples by mNGS and ascites culture. A total of 42 samples were detected pathogen positive by mNGS (Figure 1A). Among all pathogens, the positivity rates were highest in viruses (50%, 25/50), followed by bacteria (46%, 23/50) and fungi (26%, 13/50). Furthermore, gram-negative bacteria had higher positivity rates (28%, 14/50) than gram-positive bacteria (20%, 10/50), with mycobacteria the lowest positivity rates (6%, 3/50) (Figure 1A). Totally, 29 strains of bacteria, 11 strains of fungi, and 9 strains of viruses were detected by mNGS (Figure 1E). Klebsiella pneumoniae (3/50) and pseudomonas aeruginosa (3/50) were the most frequently detected bacteria; Aspergillus flavus (4/50) was the most frequently detected fungus; And human gammaherpesvirus 4 (17/50) was the most frequently detected virus.

Twenty-three patients were detected to be infected with 2 or more pathogens by mNGS (Figure 1B, C). Among them, 12 patients were positive both for bacteria and viruses; 5 patients were positive both for bacteria and fungi; 4 patients were positive both for fungi and viruses; 2 patients were positive for all three types of pathogens (Figure 1B).

As Figure 1D shows, 40 patients were only positive by mNGS; 7 patients were double negative (mNGS and ascites culture); 3 patients were double positive. The detected pathogens from the 3 double-positive patients were all bacteria, which were consistent between mNGS and ascites culture at genus level in 3 patients (100%), and were consistent at species level in 2 patients (66.7%). In addition, mNGS had short detection time than ascites culture (approximately 24 hours vs. 3-7 days).

The detailed detection results of each patient were shown in Table S1. In addition, the supplementary results of mNGS were revealed in Figure S1.

### Association between mNGS results and clinical characteristics in ESLD patients

To evaluate the clinical value of mNGS for ESLD patients with different clinical characteristics, mNGS results were then compared between these patients. As shown in Table 2, no significant difference was found in mNGS positivity rates between ESLD patients with different clinical characteristics. Moreover, the characteristics of the 42 patients with positive mNGS results were similar to those with negative mNGS results (Table S2). Furthermore, there was no significant correlation between clinical characteristics and pathogen positivity rates of mNGS (Table S3).

In addition, we compared the characteristics of ESLD patients with different numbers of pathogens detected by mNGS. Interestingly, patients with 2 or more pathogens had lower BMI (20.3 vs. 23.5, *P* = 0.049) and total cells in ascites (326.0 vs. 1182.5, *P* = 0.032), and higher platelet (110.0 vs. 68.0, *P* = 0.005) than patients with 1 or no pathogen (Table S4). Furthermore, two-variable and multivariable correlation analyses were conducted to assess the correlation between clinical characteristics and numbers of pathogens detected by mNGS. BMI, total cells in ascites, polymorphonuclear neutrophils (PMN) in ascites, platelet, and aspartate aminotransferase were correlated with numbers of pathogens with *P* values less than 0.1 in the Kendall's rank correlation analyses. But in the multivariable correlation analyses, these factors were not significantly correlated with numbers of pathogens (Table S5).

### Comparison between mNGS and conventional methods

To further assess the clinical value of mNGS, the results of mNGS and conventional methods (ascites culture and polymorphonuclear neutrophils detection) were compared (Table 3). The positive rates were greater in mNGS than in conventional methods (84% vs. 20%, *P*<0.001). The results of mNGS are considered to be “true positive” when the pathogens detected by mNGS are in the antimicrobial spectrum of the antibiotics used and the final diagnosis is peritonitis. Compared to conventional methods, mNGS had higher sensitivity (45.2% vs. 23.8%, *P*=0.039), but lower specificity (25% vs 100%, *P* = 0.010). When two of the results [mNGS, conventional methods and final diagnosis (diagnosis was made by clinicians based on guideline 7, 10)] both indicate ascites infection, the two results are defined as consistent. Furthermore, the concordance rates between mNGS results and final clinical diagnoses were similar to those between the results of conventional methods and final clinical diagnoses (42% vs. 36%, *P*=0.539).

## Discussion

This study collected ascites samples from 50 ESLD patients and performed pathogen detection using mNGS and clinical conventional methods. And the results of mNGS were compared with those of clinical conventional methods. It was found that compared to conventional methods, mNGS had significantly higher positive rates and sensitivity, broader pathogen spectrum, shorter detection time, but lower specificity. Compared with conventional methods, mNGS showed similar consistence with final diagnosis.

In this study, mNGS showed higher positive rates and broader pathogens spectrum (more detected pathogens) than conventional methods. The results were similar to those of previous studies on central nervous system infections, blood infections, lung infections, and connective tissue diseases 14-17, which suggests the good ability of mNGS in detecting all potential pathogens. Similar to previous studies on lower respiratory tract infections 18, 19, this study revealed that mixed infection was also common in peritonitis, and the most common mixed infection was bacterial-viral infection. Compared to patients with 1 or no pathogen, patients with mixed pathogens may have more severe symptoms, and therapy strategies for them may be more complicated. Through the quick and comprehensive detection of pathogens by mNGS, more appropriate antibiotic therapy strategies can be made, and thus improve the prognosis of patients with mixed infection.

Spontaneous bacterial peritonitis (SBP) is the most common type of abdominal infection 7, 10. ESLD patients with SBP have a high mortality rate. But after early and appropriate diagnosis and treatment, the mortality of these patients could be reduced from the initial over 90% to approximately 20% 20. However, in clinical practice, the ascites culture is low in positive rates and time-consuming, which makes the early diagnosis of SBP, especially the early etiological diagnosis extremely difficult. In this study, the bacterial positive rate and sensitivity of mNGS were significantly higher than those of the culture, and up to 29 potential pathogenic bacteria were detected, which was similar to the results of a previous study 21, reflecting the high application value of mNGS in identifying the pathogenic bacteria of SBP. Gram-negative bacteria were the most frequently detected bacterial group, which is in line with the results of a current epidemiological study 22. Mycobacteria infection may also cause peritonitis, and thus lead to high mortality 23. In this study, mycobacteria were detected using mNGS in 3 patients, while no mycobacterium was detected using culture. The results suggest the good clinical value of mNGS in identifying the mycobacterium infection in ascites, which may help to bring appropriate treatment and better prognosis to patients. In addition, in our study, the ascites samples from 3 patients had bacteria detected by both ascites culture and mNGS. The detected bacteria were consistent between ascites culture and mNGS at genus level in 3 patients (100%), and were consistent at species level in 2 patients (66.7%), indicating high reliability of mNGS. Moreover, there were 3 patients who had >250 × 10^6/L PMNs in ascetic fluid, but culture remained negative, and mNGS detected bacteria. They all received antibiotic therapy specific for the detected bacteria, and after therapy their peritonitis improved, which indicates good clinical value of mNGS.

In addition to bacteria, fungi are also common pathogens of peritonitis in ESLD patients. The mortality of patients with fungal peritonitis is significantly increased 24. Therefore, these patients also need timely diagnosis and treatment. In the present study, mNGS had a positive rate of 26% for fungi, while ascites culture did not detect fungi, which is similar to the results of a previous study 21. This reveals that mNGS may also contribute to the diagnosis of abdominal fungal infection in ESLD patients.

Viral infection may lead to severe diseases such as liver failure and liver cancer 25, but the current conventional detection methods do not detect the viruses that cause ascites infection. mNGS can detect all potential pathogens including viruses in samples. In this study, the positive rate of virus by mNGS was as high as 50%, suggesting that patients with ESLD may often have abdominal virus infection. However, the effect of virus infection in ascites on the prognosis of patients and whether it needs treatment remained to be further explored.

To assess the clinical value of mNGS in ESLD patients with different clinical characteristics, this study compared the mNGS positive rates between these patients. The results reveal that mNGS may have similar and high positive rates in various ESLD patients, which indicates similar clinical value in these patients.

This study detailly discussed the mNGS results in detecting pathogens of ascites in ESLD patients, and found its high clinical value. However, the application of mNGS in pathogen detection also has limitations. Since mNGS can detect all potential pathogens, including some pathogens that may not be the cause of the patient's disease (low specificity), clinicians need to comprehensively consider the patient's clinical symptoms, signs and laboratory test results when referring to the mNGS results to cautiously make the final diagnosis and guide treatment. In addition, due to the high cost of mNGS, some patients may not be able to afford it. Thus, it is necessary to reduce the cost of mNGS through technological innovation so that more patients can benefit from this technology. Moreover, considering a relatively small sample of this study, studies with a large sample are needed to verify our findings.

Based on our findings, we recommend mNGS in the following situations: 1) If the patients' economic condition is good and they provide informed consent, the ascites should be sent immediately for both mNGS and traditional cultures; 2) If the ascites are not sent for mNGS initially because of poor economic condition or other reasons, mNGS is also recommended for pathogen detection of subsequent samples in the following situations: Ⅰ) The initial cultures and other traditional methods do not detect any pathogens, and peritonitis is not improved by empirical antibiotics treatment; Ⅱ) Clinical status of patient continuously deteriorates despite of being on appropriate antimicrobials based on traditional cultures.

In conclusion, mNGS showed good ability in detecting all potential pathogens in ascites samples from ESLD patients. And compared to conventional methods, mNGS had better sensitivity in the diagnosis of peritonitis in ESLD patients with shorter detection time. Therefore, mNGS may be a good supplement for conventional methods and helpful to the early diagnosis of peritonitis, and thus improve the survival of ESLD patients.

## Supplementary Material

Supplementary figure 1 and tables 2-5.

Supplementary table 1.

Supplementary table 6.

## Figures and Tables

**Table 1:** Characteristics of patients with end-stage liver disease

Variables	Patients (n=50)
Age^#^ (years)	56.0 (18.0-76.0)
Gender	
Male (%), (n/n)	70.0 (35/50)
Female (%), (n/n)	30.0 (15/50)
BMI^#^ (kg/m²)	22.2 (14.8-34.9)
Etiologies of end-stage liver disease (%), (n/n)	
HBV	58.0 (29/50)
HCV	6.0 (3/50)
Alcohol liver disease	20.0 (10/50)
Autoimmune liver disease	10.0 (5/50)
Non-alcoholic fatty liver disease	2.0 (1/50)
Unknown	4.0 (2/50)
Comorbidities (%), (n/n)	
Gastrointestinal bleeding	12.0 (6/50)
Hepatic encephalopathy	18.0 (9/50)
Cancer (excluding liver cancer)	10.0 (5/50)
Infection (excluding peritonitis)	24.0 (12/50)
Diabetes	10.0 (5/50)
Total cells in ascites^#^ (10^6/L)	610.0 (129-151901)
PMN in ascites^#^ (10^6/L)	12.9 (1.3-4056.4)
PMN counts < 250*10^6/L (%), (n/n)	84.0 (42/50)
PMN counts ≥ 250*10^6/L (%), (n/n)	14.0 (7/50)
Unknown	2.0 (1/50)
Routine blood test	
RBC^#^ (10^9/L)	3.16 (1.16-5.22)
HB^#^ (g/L)	98.0 (41.0-156.0)
WBC^#^ (10^9/L)	4.88 (1.30-15.31)
PLT^#^ (10^9/L)	86.0 (24.0-480.0)
Liver function test	
TP^#^ (g/L)	61.4 (39.0-83.2)
ALB^#^ (g/L)	29.4 (19.8-50.5)
ALT^#^ (U/L)	19.0 (5.0-225.0)
AST^#^ (U/L)	45.5 (17.0-447.0)
TB^#^ (μmol/L)	41.4 (9.9-231.0)
DB^#^ (μmol/L)	23.7 (3.8-199.6)
PT^#^ (seconds)	17.6 (12.8-30.5)
PTA^#^ (%)	57.5 (25.0-109.0)
INR^#^	1.45 (0.95-3.01)
PCT^#^ (ng/mL)	0.18 (0.03-12.17)
PCT < 0.5 ng/mL (%), (n/n)	70.0 (35/50)
PCT ≥ 0.5 ng/mL (%), (n/n)	24.0 (12/50)
Unknown (%), (n/n)	6.0 (3/50)

^#^Presented as median (range). ALB, albumin; ALT, alanine aminotransferase; AST, aspartate aminotransferase; BMI, body mass index; DB, direct bilirubin; HB, hemoglobin; INR, international normalized ratio; PCT, procalcitonin; PLT, platelet; PMN, polymorphonuclear neutrophils; PT, prothrombin time; PTA, prothrombin activity; RBC, red blood cell; TB, total bilirubin; TP, total protein; WBC, white blood cell.

**Table 2:** Comparison of mNGS positivity rates between ESLD patients with different clinical characteristics

	All pathogens			Bacteria and fungi		
Comparisons between patients with different clinical characteristics	Positive (number)	Negative (number)	*P* value	Positive (number)	Negative (number)	*P* value
**Comparison 1**						
PMN counts < 250*10^6/L	35	7	1.000	25	17	1.000
PMN counts ≥ 250*10^6/L	6	1		4	3	
**Comparison 2**						
Culture positive	2	1	0.414	2	1	1.000
Culture negative	40	7		28	19	
**Comparison 3**						
With peritonitis symptoms	21	4	1.000	16	9	0.564
Without peritonitis symptoms	21	4		14	11	
**Comparison 4**						
PCT < 0.5 ng/mL	30	5	0.684	23	12	0.050
PCT ≥ 0.5 ng/mL	9	3		4	8	
**Comparison 5**						
HBV	24	5	0.064	19	10	0.844
HCV	1	2		1	2	
Alcohol liver disease	10	0		5	5	
Autoimmune liver disease	5	0		3	2	
Non-alcoholic fatty liver disease	1	0		1	0	
Unknown	1	1		1	1	
**Comparison 6**						
With other infection (excluding peritonitis)	12	0	0.200	8	4	0.839
Without other infection	30	8		22	16	

Chi-square test and Fisher's exact test were used for comparison. ESLD, end-stage liver disease; mNGS, metagenomic next-generation sequencing; PCT, procalcitonin; PMN, polymorphonuclear neutrophils.

**Table 3:** Comparison between final diagnosis and results of mNGS/conventional methods

	mNGS	Conventional methods	Final clinical diagnosis
Positive cases (n/n, %)	42/50, 84%	10/50, 20%	42/50, 84%
*P* value	<**0.001**		/
Sensitivity (n/n, %)	19/42,45.2%	10/42. 23.8%	/
*P* value	**0.039**		/
Specificity (n/n, %)	2/8, 25%	8/8, 100%	/
*P* value	**0.010**		/
Concordance rates between diagnosis and results of mNGS/conventional methods (n/n, %)	21/50, 42%	18/50, 36%	/
*P* value	0.539		/

Notes: conventional methods included ascites culture and PMN (in ascites) detection. The results of mNGS are considered to be “true positive” when the pathogens detected by mNGS are in the antimicrobial spectrum of the antibiotics used and the final diagnosis is peritonitis. When two of the results (mNGS, conventional methods and final diagnosis) both indicate ascites infection, the two results are defined as consistent. The chi-square test was used. mNGS, metagenomic next-generation sequencing; PMN, polymorphonuclear neutrophils.

## Abbreviations

ALB: albumin
ALT: alanine aminotransferase
AST: aspartate aminotransferase
BMI: body mass index
DB: direct bilirubin
ESLD: end-stage liver disease
HB: hemoglobin
IC: internal controls
INR: international normalized ratio
mNGS: metagenomic next-generation sequencing
PCT: procalcitonin
PLT: platelet
PMN: polymorphonuclear neutrophils
PT: prothrombin time
PTA: prothrombin activity
RBC: red blood cell
SBP: Spontaneous bacterial peritonitis
TB: total bilirubin
TP: total protein
WBC: white blood cell

## References

[1] Foreman KJ, Marquez N, Dolgert A (2018). Forecasting life expectancy, years of life lost, and all-cause and cause-specific mortality for 250 causes of death: reference and alternative scenarios for 2016-40 for 195 countries and territories. The Lancet.

[2] Health United States, 2017 (2018). With Special Feature on Mortality. Hyattsville (MD).

[3] Tapper EB, Parikh ND (2018). Mortality due to cirrhosis and liver cancer in the United States, 1999-2016: observational study. BMJ.

[4] Fernandez J, Prado V, Trebicka J (2019). Multidrug-resistant bacterial infections in patients with decompensated cirrhosis and with acute-on-chronic liver failure in Europe. J Hepatol.

[5] Lingiah VA, Pyrsopoulos NT (2021). Bacterial Infections in Cirrhotic Patients in a Tertiary Care Hospital. J Clin Transl Hepatol.

[6] Fernandez J, Piano S, Bartoletti M (2021). Management of bacterial and fungal infections in cirrhosis: The MDRO challenge. J Hepatol.

[7] Chinese Society of Infectious Diseases CMA (2022). [Expert consensus on diagnosis and treatment of end-stage liver disease complicated infection (2021 version)]. Zhonghua Gan Zang Bing Za Zhi.

[8] Li G, Sun J, Pan S (2019). Comparison of the Performance of Three Blood Culture Systems in a Chinese Tertiary-Care Hospital. Front Cell Infect Microbiol.

[9] Dever JB, Sheikh MY (2015). Review article: spontaneous bacterial peritonitis-bacteriology, diagnosis, treatment, risk factors and prevention. Aliment Pharmacol Ther.

[10] European Association for the Study of the Liver (2018). Electronic address eee, European Association for the Study of the L. EASL Clinical Practice Guidelines for the management of patients with decompensated cirrhosis. J Hepatol.

[11] Chiu CY, Miller SA (2019). Clinical metagenomics. Nat Rev Genet.

[12] Govender KN, Street TL, Sanderson ND (2021). Metagenomic Sequencing as a Pathogen-Agnostic Clinical Diagnostic Tool for Infectious Diseases: a Systematic Review and Meta-analysis of Diagnostic Test Accuracy Studies. J Clin Microbiol.

[13] Diao Z, Han D, Zhang R (2022). Metagenomics next-generation sequencing tests take the stage in the diagnosis of lower respiratory tract infections. J Adv Res.

[14] Zhang Y, Cui P, Zhang HC (2020). Clinical application and evaluation of metagenomic next-generation sequencing in suspected adult central nervous system infection. J Transl Med.

[15] Chien JY, Yu CJ, Hsueh PR (2022). Utility of Metagenomic Next-Generation Sequencing for Etiological Diagnosis of Patients with Sepsis in Intensive Care Units. Microbiol Spectr.

[16] Jin X, Li J, Shao M (2022). Improving Suspected Pulmonary Infection Diagnosis by Bronchoalveolar Lavage Fluid Metagenomic Next-Generation Sequencing: a Multicenter Retrospective Study. Microbiol Spectr.

[17] Su R, Yan H, Li N (2022). Application value of blood metagenomic next-generation sequencing in patients with connective tissue diseases. Front Immunol.

[18] Liang M, Fan Y, Zhang D (2022). Metagenomic next-generation sequencing for accurate diagnosis and management of lower respiratory tract infections. Int J Infect Dis.

[19] Tsitsiklis A, Osborne CM, Kamm J (2022). Lower respiratory tract infections in children requiring mechanical ventilation: a multicentre prospective surveillance study incorporating airway metagenomics. The Lancet Microbe.

[20] Piano S, Fasolato S, Salinas F (2016). The empirical antibiotic treatment of nosocomial spontaneous bacterial peritonitis: Results of a randomized, controlled clinical trial. Hepatology.

[21] Chen H, Zhang Y, Zheng J (2021). Application of mNGS in the Etiological Diagnosis of Thoracic and Abdominal Infection in Patients with End-Stage Liver Disease. Front Cell Infect Microbiol.

[22] Fiore M, Di Franco S, Alfieri A (2019). Spontaneous bacterial peritonitis caused by Gram-negative bacteria: an update of epidemiology and antimicrobial treatments. Expert Rev Gastroenterol Hepatol.

[23] Baldolli A, Daurel C, Verdon R (2019). High mortality in peritonitis due to Mycobacterium avium complex: retrospective study and systematic literature review. Infect Dis (Lond).

[24] Hu S, Tong R, Bo Y (2019). Fungal peritonitis in peritoneal dialysis: 5-year review from a North China center. Infection.

[25] Bunchorntavakul C, Reddy KR (2020). Epstein-Barr Virus and Cytomegalovirus Infections of the Liver. Gastroenterol Clin North Am.

