# Supplementary material for: Use of metagenomic next-generation sequencing for diagnosis of peritonitis in end-stage liver disease
Source: Int J Med Sci. 2023 Oct 9;20(13):1698–704. doi: 10.7150/ijms.89242 (PMC10620865; doi:10.7150/ijms.89242)
Supplement: Supplementary file 1 — Supplementary figure 1 and tables 2-5. [file ijmsv20p1698s1.pdf]

Use of metagenomic next-generation sequencing for diagnosis of peritonitis in end-stage liver disease

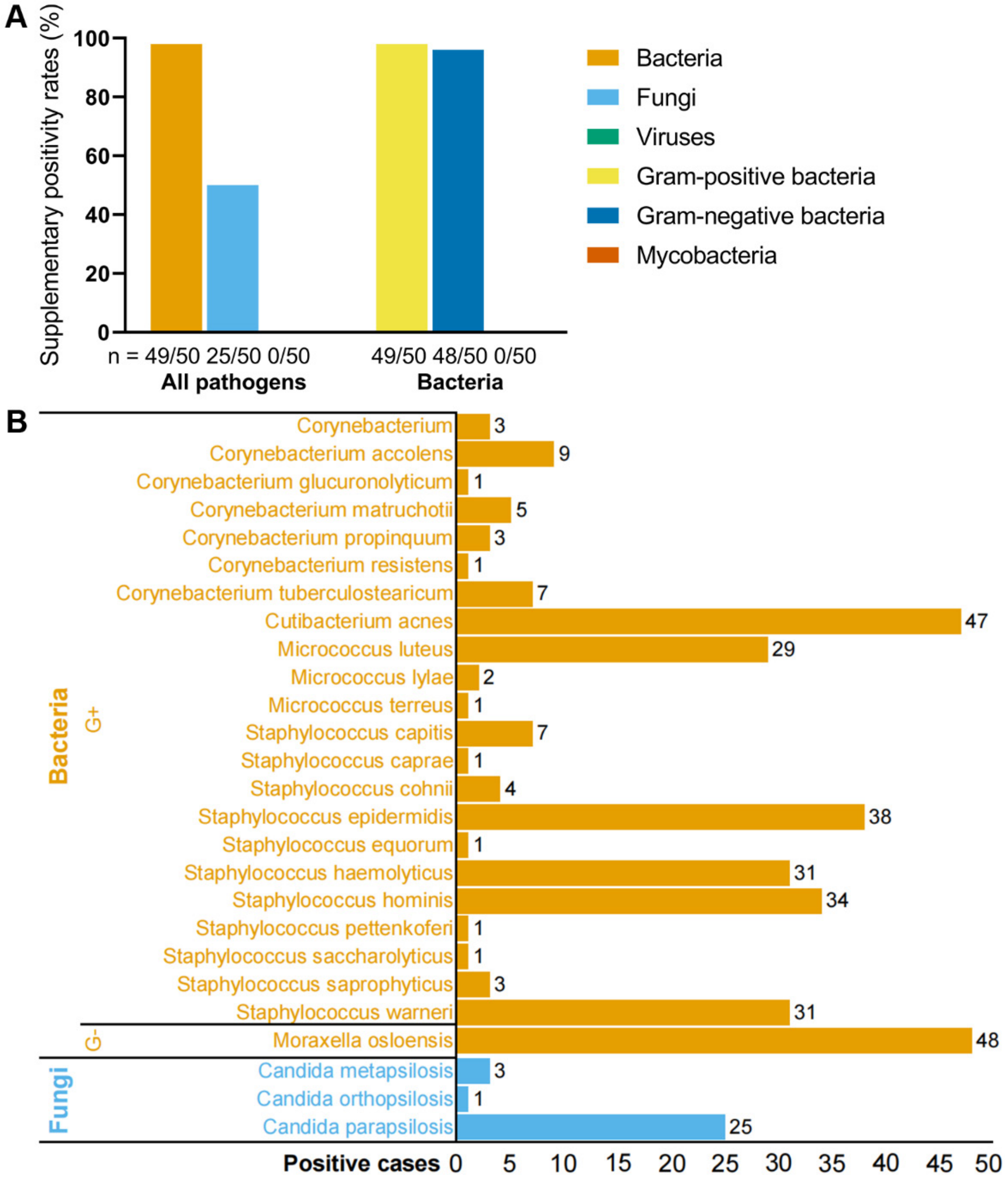

Figure S1. Supplementary detected pathogens by mNGS. (A) Supplementary positive rates of all pathogens and bacteria by mNGS in the 50 ESLD patients. **(B)** Supplementary positive cases of bacteria, fungi, and DNA viruses. mNGS, metagenomic next-generation sequencing. Supplementary mNGS results may represent the colonized/commensal microbes from patients, or environmental microbes during sample processing.

Table S2. Characteristics of ESLD patients with positive/negative mNGS results

Variables	Patients with positive mNGS results (n=42)	Patients with negative mNGS results (n=8)	*P* value
Age[#] (years)	56.0 (18.0-76.0)	51.5 (42.0-68.0)	0.427
Gender			
Male (%), (n/n)	69.0 (29/42)	75.0 (6/8)	1.000
Female (%), (n/n)	31.0 (13/42)	25.0 (2/8)	
BMI[#] (kg/m²)	23.1 (16.5-32.5)	20.7 (14.8-34.9)	0.343
Etiologies of end-stage liver disease (%), (n/n)			
HBV	57.1 (24/42)	62.5 (5/8)	1.000
HCV	2.4 (1/42)	25.0 (2/8)	0.063
Alcohol liver disease	23.8 (10/42)	0.0 (0/8)	0.289
Autoimmune liver disease	11.9 (5/42)	0.0 (0/8)	0.577
Non-alcoholic fatty liver disease	2.4 (1/42)	0.0 (0/8)	1.000
Unknown	2.4 (1/42)	12.5 (1/8)	0.297
Comorbidities (%), (n/n)			
Gastrointestinal bleeding	9.5 (4/42)	25.0 (2/8)	0.242
Hepatic encephalopathy	16.7 (7/42)	25.0 (2/8)	0.952
Cancer (excluding liver cancer)	11.9 (5/42)	0.0 (0/8)	0.577
Infection (excluding peritonitis)	28.6 (12/42)	0.0 (0/8)	0.200
Diabetes	11.9 (5/42)	0.0 (0/8)	0.577
Total cells in ascites[#] (10^6/L)	605.0 (129-151901)	1140.0 (216-135530)	0.344
PMN in ascites[#] (10^6/L)	12.9 (1.3-4056.4)	14.4 (2.7-1582.3)	0.720
PMN counts ＜ 250*10^6/L (%), (n/n)	83.3 (35/42)	87.5 (7/8)	1.000
PMN counts ⩾ 250*10^6/L (%), (n/n)	14.3 (6/42)	12.5 (1/8)	1.000
Unknown	2.4 (1/42)	0.0 (0/8)	1.000
Routine blood test			
RBC[#] (10^9/L)	3.18 (1.16-5.22)	2.72 (1.49-3.46)	0.160
HB[#] (g/L)	102.0 (41.0-156.0)	89.0 (52.0-122.0)	0.341
WBC[#] (10^9/L)	5.05 (1.83-15.31)	3.55 (1.30-6.33)	0.078
PLT[#] (10^9/L)	90.0 (25.0-480.0)	65.5 (24.0-212.0)	0.123
Liver function test			
TP[#] (g/L)	61.4 (39.0-83.2)	63.0 (44.1-71.1)	0.668
ALB[#] (g/L)	29.1 (19.8-50.5)	30.0 (24.2-44.9)	0.541
ALT[#] (U/L)	19.0 (9.0-225.0)	20.0 (5.0-163.0)	0.631
AST[#] (U/L)	47.0 (19.0-447.0)	29.0 (17.0-182.0)	0.244
TB[#] (μmol/L)	45.5 (10.0-231.0)	32.4 (12.7-84.0)	0.244
DB[#] (μmol/L)	26.8 (3.8-199.6)	16.2 (6.9-73.1)	0.315
PT[#] (seconds)	16.9 (12.8-30.5)	20.0 (13.5-27.5)	0.244
PTA[#] (%)	61.5 (25.0-109.0)	46.5 (29.0-96.0)	0.233
INR[#]	1.38 (0.95-3.01)	1.60 (1.03-2.62)	0.266
PCT[#] (ng/mL)	0.18 (0.03-12.17)	0.28 (0.05-1.93)	0.967
PCT ＜ 0.5 ng/mL (%), (n/n)	71.4 (30/42)	62.5 (5/8)	0.933
PCT ⩾ 0.5 ng/mL (%), (n/n)	21.4 (9/42)	37.5 (3/8)	0.600
Unknown (%), (n/n)	7.1 (3/42)	0.0 (0/8)	1.000

[#]Presented as median (range). The Mann–Whitney U test was used for continuous-variable comparisons. The chi-square test and Fisher's exact test were used for categorical-variable comparisons. ALB, albumin; ALT, alanine aminotransferase; AST, aspartate aminotransferase; BMI, body mass index; DB, direct bilirubin; ESLD, end-stage liver disease; HB, hemoglobin; INR, international normalized ratio; PCT, procalcitonin; PLT, platelet; PMN, polymorphonuclear neutrophils; PT, prothrombin time; PTA, prothrombin activity; RBC, red blood cell; TB, total bilirubin; TP, total protein; WBC, white blood cell.

Table S3. Correlation between clinical characteristics and positivity rates of mNGS

	r	*P*
Age (years)	0.098	0.411
Gender (male)	-0.048	0.739
BMI (kg/m^2)	0.119	0.327
Etiologies of end-stage liver disease	0.069	0.604
Comorbidities	0.127	0.375
Total cells in ascites (10^6/L)	-0.116	0.330
PMN in ascites (10^6/L)	-0.045	0.705
RBC (10^9/L)	0.169	0.153
HB (g/L)	0.116	0.327
WBC (10^9/L)	0.209	0.076
PLT (10^9/L)	0.183	0.122
TP (g/L)	-0.052	0.662
ALB (g/L)	-0.073	0.534
ALT (U/L)	0.060	0.615
AST (U/L)	0.139	0.239
TB (μmol/L)	0.140	0.234
DB (μmol/L)	0.122	0.302
PT (seconds)	-0.139	0.239
PTA (%)	0.142	0.228
INR	-0.134	0.255
PCT (ng/mL)	-0.007	0.955

Kendall's rank correlation (when one of the variables was discontinuous) was used for two-variable correlation analysis. A two-sided *P* value lower than 0.05 was considered statistically significant. r represents correlation coefficient.

ALB, albumin; ALT, alanine aminotransferase; AST, aspartate aminotransferase; BMI, body mass index; DB, direct bilirubin; HB, hemoglobin; INR, international normalized ratio; PCT, procalcitonin; PLT, platelet; PMN, polymorphonuclear neutrophils; PT, prothrombin time; PTA, prothrombin activity; RBC, red blood cell; TB, total bilirubin; TP, total protein; WBC, white blood cell.

Table S4. Characteristics of ESLD patients with different numbers of pathogens detected by mNGS

Variables	Patients with 1 or no pathogen (n=27)	Patients with 2 or more pathogens (n=23)	*P* value
Age[#] (years)	56.0 (35.0-68.0)	57.0 (18.0-76.0)	0.441
Gender			
Male (%), (n/n)	70.4 (19/27)	69.6 (16/23)	1.000
Female (%), (n/n)	29.6 (8/27)	30.4 (7/23)	
BMI[#] (kg/m²)	23.5 (14.8-34.9)	20.3 (16.5-31.1)	**0.049**
Etiologies of end-stage liver disease (%), (n/n)			
HBV	48.1 (13/27)	69.6 (16/23)	0.126
HCV	11.1 (3/27)	0.0 (0/23)	0.293
Alcohol liver disease	29.6 (8/27)	8.7 (2/23)	0.136
Autoimmune liver disease	3.7 (1/27)	17.4 (4/23)	0.256
Non-alcoholic fatty liver disease	3.7 (1/27)	0.0 (0/23)	1.000
Unknown	3.7 (1/27)	4.3 (1/23)	1.000
Comorbidities (%), (n/n)			
Gastrointestinal bleeding	11.1 (3/27)	13.0 (3/23)	1.000
Hepatic encephalopathy	29.6 (8/27)	4.3 (1/23)	0.051
Cancer (excluding liver cancer)	11.1 (3/27)	8.7 (2/23)	1.000
Infection (excluding peritonitis)	18.5 (5/27)	30.4 (7/23)	0.325
Diabetes	7.4 (2/27)	13.0 (3/23)	0.850
Total cells in ascites[#] (10^6/L)	1182.5 (216-151901)	326.0 (129-44020)	**0.032**
PMN in ascites[#] (10^6/L)	16.9 (2.7-1582.3)	8.5 (1.3-4056.4)	0.054
PMN counts ＜ 250*10^6/L (%), (n/n)	81.5 (22/27)	87.0 (20/23)	0.889
PMN counts ⩾ 250*10^6/L (%), (n/n)	14.8 (4/27)	13.0 (3/23)	1.000
Unknown	3.7 (1/27)	0.0 (0/23)	1.000
Routine blood test			
RBC[#] (10^9/L)	2.97 (1.16-5.22)	3.27 (1.57-5.07)	0.164
HB[#] (g/L)	99.0 (41.0-156.0)	96.0 (44.0-148.0)	0.508
WBC[#] (10^9/L)	4.82 (1.30-15.31)	5.17 (1.83-13.4)	0.527
PLT[#] (10^9/L)	68.0 (24.0-299.0)	110.0 (31.0-480.0)	**0.005**
Liver function test			
TP[#] (g/L)	63.4 (42.3-83.2)	59.6 (39.0-81.5)	0.122
ALB[#] (g/L)	29.8 (19.8-44.9)	28.7 (23.8-50.5)	0.969
ALT[#] (U/L)	19.0 (5.0-225.0)	19.0 (9.0-114.0)	0.508
AST[#] (U/L)	37.0 (17.0-294.0)	56.0 (19.0-447.0)	0.090
TB[#] (μmol/L)	40.8 (10.0-231.0)	42.0 (9.9-209.7)	1.000
DB[#] (μmol/L)	23.8 (3.8-199.6)	23.5 (4.6-114.9)	0.853
PT[#] (seconds)	18.6 (13.5-29.9)	16.2 (12.8-30.5)	0.386
PTA[#] (%)	52.0 (26.0-96.0)	66.0 (25.0-109.0)	0.386
INR[#]	1.56 (1.03-2.92)	1.31 (0.95-3.01)	0.397
PCT[#] (ng/mL)	0.18 (0.03-12.17)	0.20 (0.05-3.48)	0.948
PCT ＜ 0.5 ng/mL (%), (n/n)	74.1 (20/27)	65.2 (15/23)	0.496
PCT ⩾ 0.5 ng/mL (%), (n/n)	25.9 (7/27)	21.7 (5/23)	0.730
Unknown (%), (n/n)	0.0 (0/27)	13.0 (3/23)	0.181

[#]Presented as median (range). The Mann–Whitney U test was used for continuous-variable comparisons. The chi-square test and Fisher's exact test were used for categorical-variable comparisons. ALB, albumin; ALT, alanine aminotransferase; AST, aspartate aminotransferase; BMI, body mass index; DB, direct bilirubin; ESLD, end-stage liver disease; HB, hemoglobin; INR, international normalized ratio; PCT, procalcitonin; PLT, platelet; PMN, polymorphonuclear neutrophils; PT, prothrombin time; PTA, prothrombin activity; RBC, red blood cell; TB, total bilirubin; TP, total protein; WBC, white blood cell.

Table S5. Correlation between clinical characteristics and numbers of pathogens detected by mNGS

	r	*P*	*β*	*P*
Age (years)	0.092	0.441		
Gender (male)	-0.009	0.951		
BMI (kg/m^2)	**-0.239**	**0.049**	-0.270	0.137
Etiologies of end-stage liver disease	-0.122	0.359		
Comorbidities	0.084	0.559		
Total cells in ascites (10^6/L)	**-0.255**	**0.032**	0.118	0.439
PMN in ascites (10^6/L)	**-0.229**	**0.054**	0.127	0.477
RBC (10^9/L)	0.164	0.164		
HB (g/L)	0.078	0.508		
WBC (10^9/L)	0.075	0.527		
PLT (10^9/L)	**0.332**	**0.005**	0.167	0.260
TP (g/L)	-0.183	0.122		
ALB (g/L)	-0.005	0.969		
ALT (U/L)	0.079	0.508		
AST (U/L)	**0.200**	**0.090**	0.172	0.257
TB (μmol/L)	0.000	1.000		
DB (μmol/L)	-0.022	0.853		
PT (seconds)	-0.102	0.386		
PTA (%)	0.103	0.386		
INR	-0.100	0.397		
PCT (ng/mL)	-0.008	0.948		

Kendall's rank correlation (when one of the variables was discontinuous) was used for two-variable correlation analysis. When the *P* value of a variable is lower than 0.1 (**bold**), this variable was included in multivariate linear regression analysis (enter method) for the further analysis. A two-sided *P* value lower than 0.05 was considered statistically significant. r represents correlation coefficient, and *β* represents the standardized regression coefficient.

ALB, albumin; ALT, alanine aminotransferase; AST, aspartate aminotransferase; BMI, body mass index; DB, direct bilirubin; HB, hemoglobin; INR, international normalized ratio; PCT, procalcitonin; PLT, platelet; PMN, polymorphonuclear neutrophils; PT, prothrombin time; PTA, prothrombin activity; RBC, red blood cell; TB, total bilirubin; TP, total protein; WBC, white blood cell.
